# Professor Haowen Xu: The founder of exercise biochemistry in China

**DOI:** 10.1007/s13238-019-00663-z

**Published:** 2019-10-21

**Authors:** Wei Gong, Yijing Shen, Jiaqi Bao, Yike Ying, Han Zhou, Zhifeng Wu

**Affiliations:** 1 Zhejiang Normal University, Jinhua, 321004 China; 2 East China Normal University, Shanghai, 200062 China

**Figure 1:**
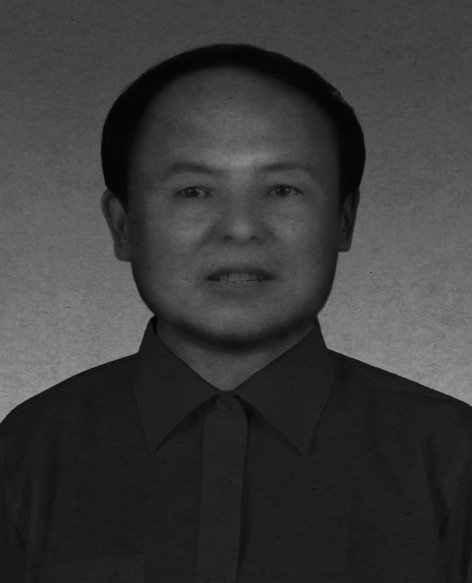
Professor Haowen Xu

Professor Haowen Xu (许豪文, 1935–2004) was a famous sports scientist and the founder of Exercise Biochemistry in China (Fig. 1). He dedicated his life to the study of sports science and made tremendous contributions to the formation and growth of the field of Exercise Biochemistry in China. He was the first to use theories of exercise biochemistry and sports medicine to guide athletes’ training and competition in China (Yu, 2006).

Prof. Xu was born in 1935 to a family of scholars in Nanjing, China. While he was a child, Prof. Xu was an accomplished student and developed a deep interest in medicine. In 1959, Prof. Xu received his bachelor’s degree as an outstanding graduate from the Department of Medicine and Therapeutics at Shanghai No. 1 Medical College (now known as Shanghai Medical College of Fudan University). Prof. Xu responded to a national call for the development of sports science and entered the Research Institute of Sports Science, part of the Physical Culture and Sports Commission of the People’s Republic of China (PRC) (now known as China Institute of Sports Science). There, he became involved in the brand-new field of real-time monitoring of athletes’ training. During this time, Prof. Xu, in cooperation with Tianle Yang (杨天乐) and Xiaomei Qin (秦晓梅), studied the variation of urine 17-hydroxycorticosteroid (17-OH), which was affected by different training loads and emotional stress in the 48 h following exercise. In 1978, Prof. Xu was transferred to the Shanghai Research Institute of Sports Science. There he was responsible for the project *Biochemical Assessment of Exercise Dose of Sprinters* and reported several important findings. During this period, Prof. Xu was awarded the Fourth-Class Prize for Scientific and Technology Progress in Sports by the Physical Culture and Sports Commission of the PRC and the Third-Class Prize for Scientific and Technology Progress of Shanghai. In 1984, Prof. Xu continued his research at East China Normal University (ECNU). After that, he was successively appointed as the deputy director of the Department of Physical Education, the deputy director of the Research Institute of Sports Science, and a member of the Academic Degree Committee and the Professional Title Evaluation Committee. As an academic leader at ECNU, Prof. Xu made significant positive contributions to the construction and development of the physical education curriculum. Given Prof. Xu’s outstanding achievements, he was awarded the Special Government Allowances of the State Council in 1992.

During the 1980s, Prof. Xu published several high-quality research articles in academic journals such as *China Sports Science*, *Journal of Physical Education*, *Sports* & *Science*, among others. In 1981, Prof. Xu explored the variations in the laws of Serum Creatine Phosphate Kinase (SCPK) and Serum Urea Nitrogen (SUN) resulting from different exercise intensities, durations, and types in a real state rather than laboratory conditions, which represented a world-wide breakthrough in this area (Xu et al., 1981). In 1992, Prof. Xu compared the activity of lipofuscin and glutathione S-transferase (GST) after swimming for 320 min in a rat model. This research proved that the activities of lipofuscin as well as GST decreased in the heart, skeletal muscle, and liver after swimming, which provided more detail regarding the interactions between free radicals and cellular zymoprotein and structural proteins during exercise (Ding et al., 1982). During the early 21st century, Prof. Xu focused on the relationships between exercise, insulin receptors, signal transduction proteins. His research revealed that exercise resulted in the improvement of skeletal muscle insulin effects and insulin signal transduction ability, which were accompanied by decreases in the expression of IRS-1/2. This work helped show the great potential for exercise training as a therapeutic treatment for type II diabetes (Sun and Xu, 2002).

Prof. Xu was also concerned with public health throughout his life. In the early 1980s, by monitoring enzyme changes in athletes during exercise, Prof. Xu discussed the rationality of exercise dose and shared his results with the public (Xu et al., 1983). In 1986, he found that the concentration of plasma testosterone increased as boys entered adolescence. In addition, androgenic hormones promoted the development of boys’ exercise capacity, which promoted further secretion of androgenic hormones. In contrast, the level of androgenic hormone remained basically unchanged in girls as they entered adolescence, which helped to explain differences in exercise capacity between adolescent boys and girls. This important revelation laid the theoretical foundation for different gender groups participating in different forms of physical exercise (Xu, 1986). Since the 1990s, there has been a fervent belief that participating in sports could delay human aging and decrease causes of increased morbidity, such as tumors. In an attempt to rectify this one-sided view, Prof. Xu released a paper entitled *Rehabilitation and Immunity of Geriatric Diseases*, and made the point that excessive exercise could lead to unfavorable consequences, such as increasing the incidence of infection, enhancing the sensitivity of tumor onset, or aggravating a disease’s course. Therefore, he proposed that people should choose reasonable exercises according to their personal situation (Xu, 1993).

**Figure 2:**
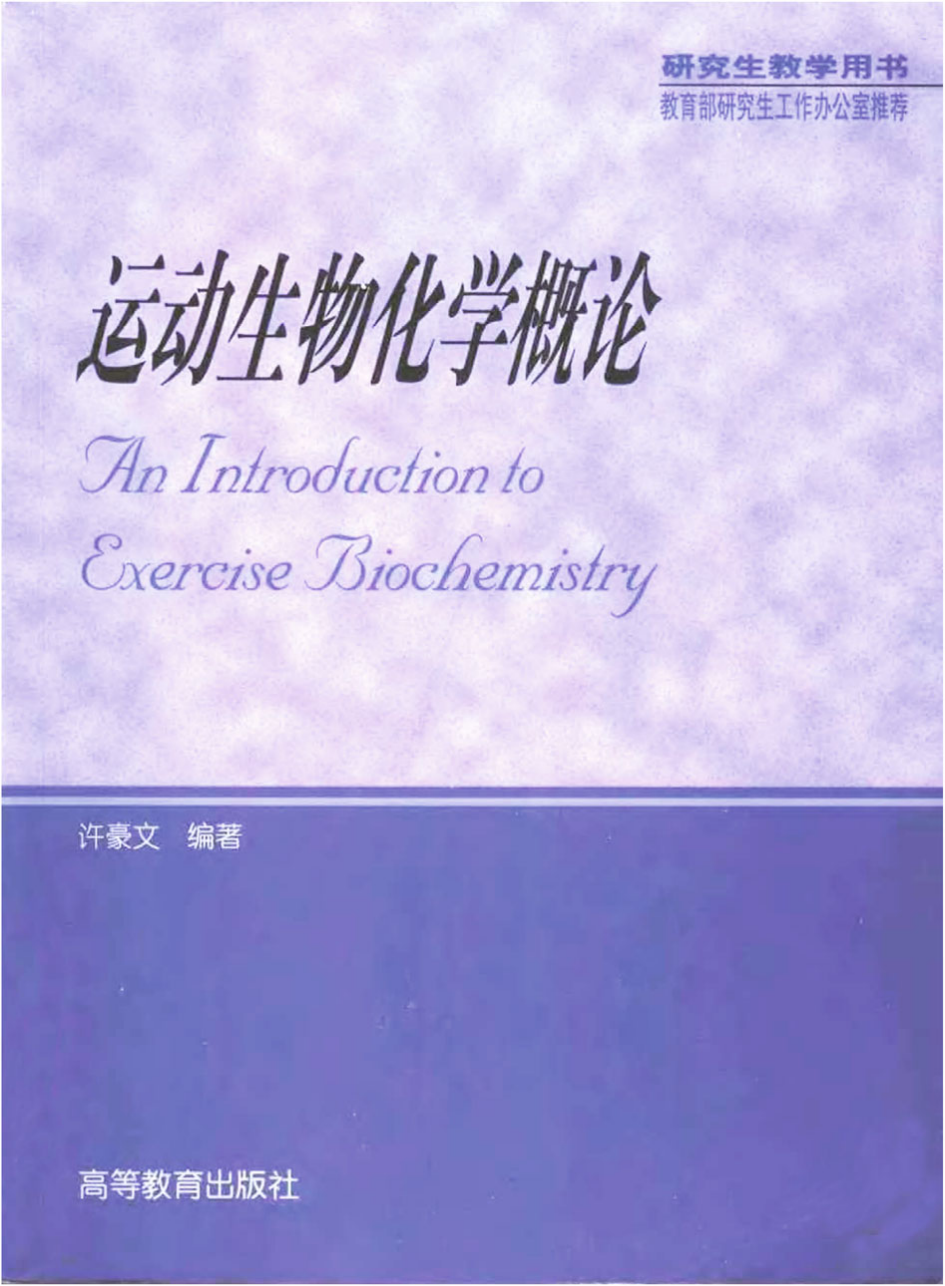
An introduction to exercise biochemistry

**Figure 3:**
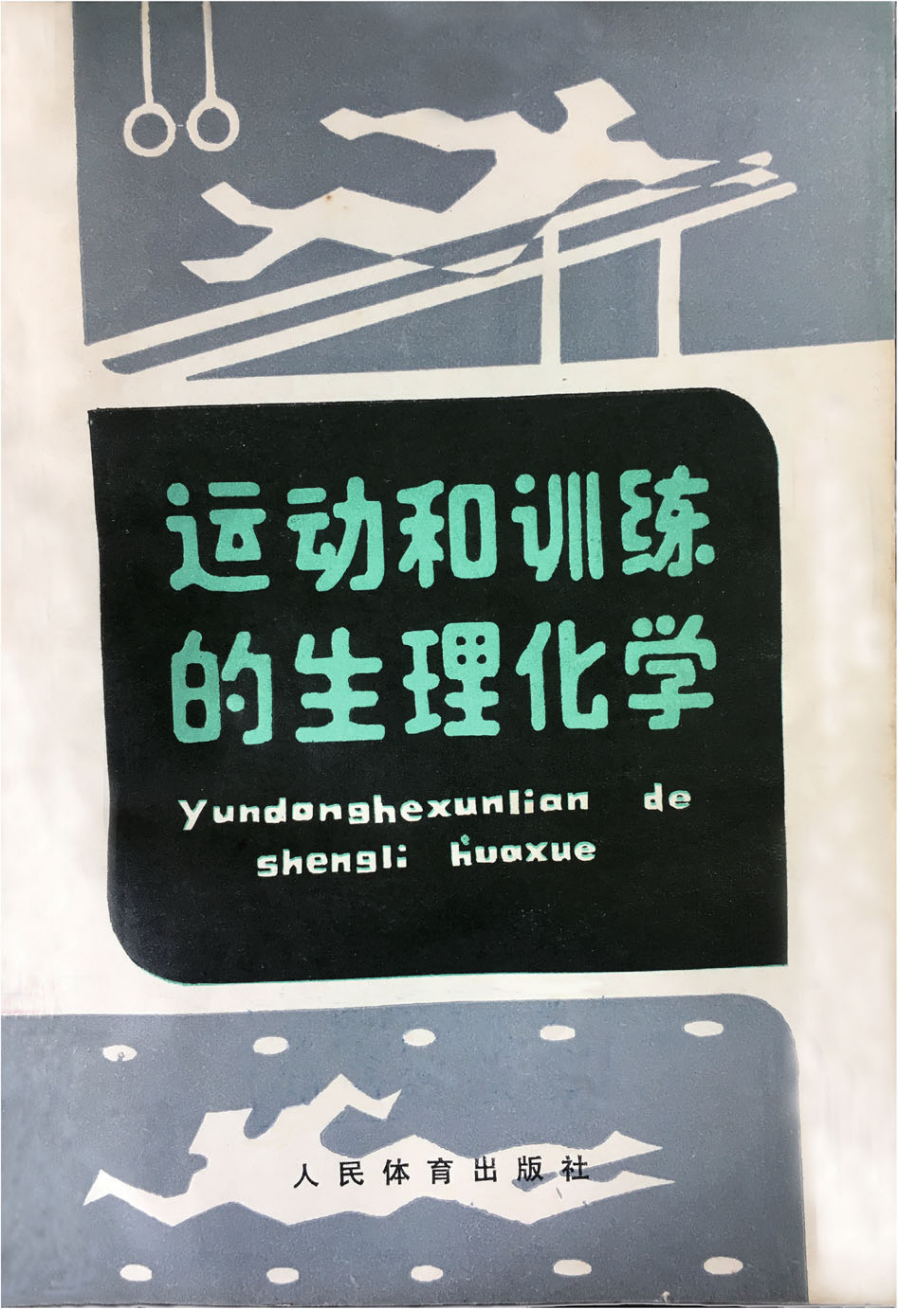
Physiological chemistry of exercise and training

Prof. Xu dedicated his entire life to the field of exercise biochemistry and contributed to more than ten monographs, such as *An Introduction to Exercise Biochemistry*, *Human Physiology*, and *Sports Medicine*, among others. *An Introduction to Exercise Biochemistry*, published in 2001, was the most representative monograph written by Prof. Xu independently (Fig. 2). This book comprehensively introduced the laws of substance metabolism and energy metabolism in the human body during exercise, as well as sports fatigue, drug abuse in competition, and more. It was recommended as a Postgraduate Teaching Book by the Postgraduate Working Office of the Ministry of Education in China and was the only such book within the field of physical education (The Postgraduate Working Office of the Ministry of Education in China, 2000). Prof. Xu also played an energetic role in introducing advancements and achievements made abroad to China. For example, he consistently recommended dozens of foreign research papers on sports fatigue in the journal *Shanxi Sports Science and Technology* starting in 1989. Additionally, Prof. Xu co-translated the conference proceedings of the First International Physiological Chemistry Seminar on Sports Training in a collection entitled *Physiological Chemistry of Exercise and Training*, with Weiquan Feng (冯炜权), Ming Hua (华明), Kuisheng Yang (杨奎生), and Bubiao Wang (王步标) (Fig. 3).

Prof. Xu enjoyed a distinct status in the field of sports science and exercise biochemistry in China. He was elected as the director of the China Sports Science Society, an editor of the *China Journal of Sports Medicine*, an honorary director of the Chinese Association of Rehabilitation Medicine, as well as a member of the Discipline Evaluation Group of the Academic Degrees Committee of the State Council. In addition, Prof. Xu was also an adjunct professor and scientific training consultant at Beijing Sports Normal College (now known as Capital University of Physical Education and Sports), Wuhan Sports University, Guangzhou Municipal Sports Work Brigade (now known as Guangzhou Polytechnic of Sports), and Soochow University. Despite his outstanding achievements and standing within his field, Prof. Xu never regarded himself as a so-called “academic authority” and always insisted on academic freedom and sharing achievements with young scholars, promoting an atmosphere of creativity and academic prosperity.

Prof. Xu placed great importance on instruction within his discipline and the cultivation of talent, and was one of the first tenured professors at ECNU (The School of Sports and Health of East China Normal University, 2015). Due to Prof. Xu’s efforts, ECNU established the first master’s program in exercise biochemistry (now renamed as kinesiology) in 1986 and the first doctoral program in exercise biochemistry in 1993 (Yu, 2006). In total, he mentored and cultivated more than 60 master’s students and 20 doctoral students at ECNU. Prof. Xu and his students worked together to overcome many difficulties in the field of exercise biochemistry, and became a significant academic force in the field of sports science in China. Professor Shuzhe Ding (丁树哲) of ECNU was Prof. Xu’s first master’s student, whose main research interests now are exercise adaptation and mitochondrial signal control. One of his outstanding contributions was being the first person in the world to observe super oxygen free radicals (O_2_^−^) in rat myocardia after fatiguing exercise using Electron Spin Resonance (ESR) (Ding et al., 1991). He also reported other important findings about the effects of exercise on the structure and function of mitochondrial membranes, and the influences of aerobic training on mitochondrial DNA (mtDNA) and mitochondrial NO synthesis (Feng et al., 2006). Associate Professor Gang Zhou (周刚) of the Physical Education Institute at Hunan University was Prof. Xu’s last doctoral student. His research in exercise biochemistry focuses on exercise-induced oxidative stress, the mechanisms of sports fatigue, and physical fitness. His research results have been published in domestic and foreign journals and include *The Effects of Growth Hormone Administration on the Circulation Level of ghrelin and IGF-I of the Trained Rats* (Zhou et al., 2009), and *Ascorbate protects against vascular leakage in cecal ligation and puncture-induced septic peritonitis* (Zhou et al., 2012).

Prof. Xu’s remarkable achievements were made possible by the support and example set by his family. Prof. Xu’s father, Professor Shijin Xu (许世瑾), was the founder of life statistics and health statistics in China. He devoted his attention to the health problems of other people throughout his life and adopted the methods of medical statistics to study the causes of death among Chinese residents (School of Public Health Shanghai Medical University, 1988). In 1935, he built the earliest reporting system for infectious diseases and parasitic diseases in China, with 204 hospitals nationwide. Consequently, the incidence and geographical distribution of 19 different kinds of diseases were investigated and counted, which provided important information for the prevention and control of infectious diseases and parasitic diseases in China (Xu and Ge, 1937). Prof. Xu’s wife, Professor Honglyu Zhou (周红律), was also a well-known exercise biochemistry expert. Using advanced technology, the couple carried out multiple studies, such as osteoporosis, vitamin K and exercise. This study revealed that intense aerobic exercise had a negative impact on bone mineral density, and that lack of vitamin K could lead to a decline of calcium-binding capacity in osteocalcin, which helped to begin to interpret the relationships between osteoporosis, vitamin K, and exercise (Xu and Zhou, 2003).

Sadly, Prof. Xu passed away in Shanghai in December 2004. He was a ground-breaking exercise biochemist and made many influential contributions to the development of sports science in China, including academic research, disciplinary development and most importantly, the cultivation of young talents. Prof. Xu was a rigorous scholar with remarkable achievements and a modest, easy-going educator who was willing to evangelize his field. He is worthy of appreciation and will serve as a model for the next generation of scientific researchers.
